# Experiences of foot and ankle mobilisations combined with home stretches in people with diabetes: a qualitative study embedded in a proof-of-concept randomised controlled trial

**DOI:** 10.1186/s13047-022-00512-z

**Published:** 2022-01-29

**Authors:** Vasileios Lepesis, Jonathan Marsden, Joanne Paton, Alec Rickard, Jos M. Latour

**Affiliations:** 1 School of Health Professions, Faculty of Health, University of Plymouth, Plymouth, PL4 8AA UK; 2 School of Nursing and Midwifery, Faculty of Health, University of Plymouth, Plymouth, UK

**Keywords:** Diabetes, Physiotherapy, Adherence, Barriers, Exercise, Mobilisations

## Abstract

**Background:**

The benefits of exercise and staying active are widely reported in the literature, however adherence and engagement with exercise amongst people with long-term illness and diabetes is poor. Physiotherapy aims to promote independence and physical activity using a range of strategies, including manual therapy and education/advice on exercises. However, low adherence impacts negatively on treatment outcomes. In this study, the practicality of physiotherapy interventions in patients who participate in a proof-of-concept (PoC) randomised controlled trial (RCT) will be considered.

**Aim:**

To explore the experiences of people with diabetes who received an intervention package of foot and ankle mobilisations combined with home stretches for a 6-week period.

**Design:**

An embedded qualitative study in a proof-of-concept RCT using semi-structured interviews and thematic analysis.

**Participants:**

Purposive sample of 16 participants (mean age 73 years) with a diagnosis of diabetes (mean duration 13.4 years) were recruited.

**Results:**

Analysis revealed seven themes informing the adherence and non-adherence to the exercise intervention. Themes describing the positive experiences were: 1) support from others to do the exercises; 2) psychological factors to motivate exercise adherence; 2) physical factors contributing to exercise adherence; 4) acceptability of home exercises during and beyond the study. Other themes described barriers: 5) social factors that contributed to exercise disengagement; 6) emotional limitations that influence exercise avoidance; 7) physical circumstances that made exercise participation burdensome. Themes highlighted positive influences by physiotherapists, the motivation of doing exercises while participating in a study, improving the perceived range of motion in their foot and ankle and reducing discomfort in these joints whilst being more active with daily activities.

**Conclusion:**

Our findings highlighted that the intervention of foot and ankle mobilisations combined with home stretches is feasible for study participants. Psycho-social support, self-efficacy, and physiotherapy support are motivational to adhere to the study intervention and might contribute to the success of a full-scale RCT.

**Supplementary Information:**

The online version contains supplementary material available at 10.1186/s13047-022-00512-z.

## Introduction

In 2019, the International Diabetes Federation estimated that 463 million adults were living with diabetes; this number is predicted to rise to 700 million adults by 2045 (IDF Diabetes Atlas, 2019). On any given day in the United Kingdom (UK), the National Diabetes Foot care Audit reports that approximately 64,000 people have an active foot ulceration (Digital, 2018).). In addition, the 5-year mortality rate after diabetic foot ulcer onset is ranges from 43 to 55% [20, 38]. Amongst other risk factors, reductions in ankle range of movement (ROM) and increases in peak plantar pressures are thought to contribute to the likelihood of foot ulcerations [19, 23, 29, 33, 39].

The importance of establishing normal walking kinematics and ankle ROM during the stance phase of gait in order to minimise the production of high PPPs has been determined [28, 41]. Interventions used to increase ankle ROM include ankle mobilisations [13, 49, 52] and stretches [43, 53]. These type of interventions which aim to address the biomechanical deficits of the foot and ankle, place more emphasis on active preventative measures rather than the traditional paradigms of diabetic foot management i.e. callus debridement and off-loading with footwear/insoles [40]. Active interventions also promote the notion of self-management which is thought to be more sustainable for adults living with long-term illness like diabetes. This becomes more urgent considering the suggestion by Imperatore et al. (2012) who estimated, that by the year 2050, there will be a 49% increase of people under the age of 20 diagnosed with type II diabetes. Therefore, engagement with active interventions and the need of self-care beyond the medical treatment will become fundamental in the years to come.

Physical activity and its different types of exercise such as aerobics, strengthening, stretching and balance exercises falls under the umbrella of self-management. Physical activity with a primary link to aerobic exercises has shown to contribute to the prevention [21] or delay in the development of long-term complications of type II diabetes by having a positive effect on glycaemic control [5, 16]. However, research suggests that people with diabetes are usually less active and engage less in physical activity than people without diabetes [30, 50]. Defining adherence to exercise can prove challenging with a recent systematic review reporting that a clear definition does not exist [2]. A potential reason for this could be the diversity of parameters used in the literature to measure adherence such as frequency of exercises, accuracy and quality. A definition of adherence widely accepted in the literature is the one provided by the World Health Organisation (2003), which states that adherence, as used in chronic disorders, is the degree to which a person’s behaviour with regard to implementing lifestyle changes (i.e. exercise), corresponds with agreed recommendations. The value of satisfactory adherence also varies in the literature, with 80% being suggested as a reasonable threshold [2]. Adherence to exercise programmes in people with diabetes varies between 10 and 80% [35].

Participants were recruited as part of the intervention group of a PoC RCT (https://clinicaltrials.gov/ct2/show/NCT03195855). The aim of the RCT was to assess whether ankle and big toe joint mobilisations and home program of stretches in people with diabetic peripheral neuropathy improves joint range of motion and reduces forefoot peak plantar pressures. Participants were randomly assigned to the intervention (*n* = 31) or control group (*n* = 30). The intervention was a 6-week programme of ankle and big toe joint mobilisations by a physiotherapist and home stretches. The control group received usual care by podiatry interventions. The aim of this embedded qualitative study was to explore the experiences of the study participants to understand how the participants experienced the intervention to inform a full-scale RCT.

## Methods

This study adopted a qualitative design with semi-structured interviews and thematic analysis which was embedded within a PoC RCT. This method offers flexibility to capture rich and meaningful data [6] to inform a full-scale RCT. A critical realist paradigm positioned between positivism and constructivism was chosen, as this reflects the author’s assumptions that the world as we understand it is constructed from our perspectives and experiences (Willig, 2013). The COnsolidated criteria for REporting Qualitative research checklist was used to report the findings of this study [51].

### Participants and recruitment

Participants in the intervention group of the PoC RCT were asked to undertake home stretches for a period of 6 weeks. The physiotherapist demonstrated the stretches to the participants and a leaflet explaining the stretches was also supplied (Electronic Supplement Material 1). To optimise adherence [12], a weekly structured diary (Electronic Supplement Material 2) was given to the participants and asked to complete the diary after every exercise. A week before their treatment was concluded (week 5), the physiotherapist provided participants an information sheet and invitation letter to participate in the embedded qualitative study. The following week, participants were asked whether they would be interested in taking part. If they agreed, the chief investigator contacted them by telephone for further verbal information and answer any questions. An interview was arranged after written informed consent was provided.

The selection of study participants was based on the purposive sampling strategy [37]. Participants were identified and selected based on their experiences to the intervention with the aim of capturing a wide range of perspectives relating to the prescribed home stretches. It was agreed that the participants’ record of adherence rates was based on their returned exercise diaries. Diaries were received from 96% of participants (30 out of 31). This information was used to detect and select participants for the interview, who either demonstrated adherence or non-adherence trends to home stretches. The overall aim was to compare and contrast and to identify similarities and differences between the adherent and the non-adherent groups [32]. However, the majority of participants in the intervention group, seemed to adhere to the stretches with an average of 83.3% (range 52.4–114.3%). Therefore, the decision was made to recruit 16 participants. During the analyses of the final two interviews, we experienced that no new main themes emerged, hence the authors assumed that adequate levels of data saturation was achieved [25]. No repeat interviews were carried out.

### Interviews

Semi-structured interviews were held at the outcome measures collection appointment of the participants in week 18 of the study. An interview guide was developed based on the literature and the proof-of-concept RCT The interviews lasted an average of twenty-minutes and were audio recorded whilst in a quiet room in the gait laboratory without other persons. The authors searched the literature to identify previous trends and themes associated with reduced exercise adherence (exercise routine, self-efficacy mood). Various themes were identified including overuse injuries and lack of motivation [36], monitoring [11], physical problems and inconvenience [45]. Participants were asked to share their experiences related to the home stretches and their thought processes for carrying out these exercises.

During the first three interviews, a phenomenon was observed by the interviewer (VL) that prompted a minor modification of the interview guide. Whilst participants were asked to share their experiences in participating in the RCT, they tended to express their feelings and thoughts of receiving physiotherapy mobilisations in their foot and ankle (manual therapy arm of intervention package). Following this, another question was included in the interview guide regarding the physiotherapist role and the mobilisations that took place in the foot and ankle (Electronic Supplement Material 3).

### Data analysis

Data was analysed using the 6-step process of thematic analysis (Braun and Clark, 2006). In the first step (familiarising), data was transcribed, and the researcher (VL) immersed himself in the data by reading and rereading the scripts. During this step, the researcher developed a deeper understanding of the data by searching and making notes of patterns/ideas. The second step (coding) involved the manual production of interesting codes across the whole data set. The third step (theme searching) carried out by two researchers (VL, JML), involved the interpretive stages of the data by collating codes into sub-themes. A visual thematic map was developed to help the researchers unpick the relationship between codes and sub-themes and divided these further into different themes. The fourth step (theme reviewing) involved the discussion between the two researchers (VL, JML) and a debate with two other researchers (JM, JP) to refine the themes in order to accurately reflect the meaning of the data. The fifth step (theme defining and naming) was a continuation of the previous step aimed to refine and define the final themes by including their descriptors (VL, JML, JM, JP). The final step (report producing) involved the final analysis which is presented in this paper.

### Rigour and trustworthiness

The chief investigator of the PoC RCT was also the interviewer (VL). The interviewer was male and worked as a Physiotherapy lecturer and a PhD student. Prior to the study he received formal training at Masters Level in qualitative research methods. The interviewer had no relationship with the participants prior to the commencement of the study. The participants had met the interviewer during the collection of baseline and follow-up outcome measures sessions and were familiar with the aims of the study. The interviewer was blinded to the group of exercise (i.e. adherent versus non-adherent group) the participants’ belonged to.

### Ethics

Ethical approval was granted by the Faculty Research Ethics Committee of the University of Plymouth (Ref: 17/18–866). The study protocol (IRAS, project ID: 228115) also received approval from NHS Health Research Authority and South West - Exeter Research Ethics Committee (Ref:17/SW/0170). Informed written consent was obtained from all the participants.

## Findings

Totally, 16 participants were interviewed: 14 males and 2 females (Table 1). Age of participants was between 60 and 86 years and all except one participant were diagnosed with Type II diabetes with a duration of diabetes between three and 28 years.

**Table 1:** Participant characteristics

Participant no	Gender	Age	BMI	Type of diabetes	Years of diabetes
1	M	76	34	Type 2	10
2	M	68	28	Type 2	10
3	M	86	28	Type 2	10
4	M	78	39	Type 2	10
5	M	79	30	Type 2	28
6	M	73	62	Type 2	6
7	M	75	31	Type 2	27
8	F	67	31	Type 2	20
9	M	75	34	Type 2	8
10	M	69	31	Type 2	17
11	M	77	32	Type 2	3
12	F	68	27	Type 2	10
13	M	73	38	Type 2	11
14	M	60	27	Type 2	6
15	M	74	27	Type 1	28
16	M	70	35	Type 2	11

*BMI* Body Mass Index

Analysis of the data revealed seven themes describing the adherence and non-adherence to the intervention of the proof-of-concept RCT. The adherence related theme included four subthemes: 1) Support from others to do the exercises; 2) Psychological factors to motivate exercise adherence; 3) Physical factors contributing to exercise adherence; 4) Acceptability of home exercises during and beyond the study. The non-adherence themes were: 5) Social factors that contributed to exercise disengagement; 6) Emotional limitations that influence exercise avoidance; 7) Physical circumstances that made exercise participation burdensome (Table 2).

**Table 2:** Themes, subthemes and quotations

Themes	Subthemes	Quotations
Support from others to do the exercises	Family support by reminding benefits of exercise	*“I used to do it when my wife said have you done your exercises today … so I really needed prompt to be reminded (P4)*
	Support from physiotherapist by positive motivation	*“It wasn’t difficult, it was rather pleasant, having the physiotherapy treatment here every week it was great, quite relaxing” (P14)*
Psychological factors to motivate exercise adherence	Moral obligation to do the exercises being a study participant	*“My feeling is that once, my philosophy in life always been, if you start something you must finish it. And that’s in any facet in life, if you start something you must finish it” (P3)*
	Contributing to research and helping future people with diabetes	*“I found participating in this research very useful and I just want to help other people and want to help me as well” (P5)*
	Gaining confidence and seeing the benefits of being study participants	*“I have noticed my ankles are freer, now at home I will walk through the hallway without my walking stick, I haven’t been doing that for a long time” (P12)*
Physical factors contributing to exercise adherence	Exercises improved mobility and flexibility in foot and ankle joints	*“Since doing the exercises, I have noticed big improvements in my movements, it has eased off the stiffness in my ankles and big toes” (P14)*
	Physiotherapy visits helped foot and ankle mobility	*I feel having the physiotherapy was making my joints feel better, not so tight, less restriction in my ankle joints” (P11)*
	Exercises and Physiotherapy visits helped to reduce pain experiences	*“I found the physio sessions so helpful, so pain relieving especially when he was pulling my leg” (P5)*
	Exercises and Physiotherapy improve participation in Activities of Daily Living	*“Bending the foot is much easier now, like picking things from the floor, or climbing up the stairs or walking a long distance” (P11)*
Acceptability of home exercises during and beyond the study	Instruction for stretches were easy to understand and exercise diary easy to complete	*“The stretches were easy to understand and had some pretty pictures to tell me what I was meant to do anyway” (P8)*
	Home stretches don’t take long to complete	*“It wasn’t taking me very long to do the exercises so that didn’t put me off” (P11)*
	Stretches were enjoyable to do	*“Stretches became* *more enjoyable, the more I did them” (P10)*
	Continue with stretches beyond study	*“I will indefinitely carry on doing them every morning, they have improved my walking and my confidence” (P15)*
Social factors that contributed to exercise disengagement	Insufficient time and lack of routine to do the home stretches	*“Not doing them as often … .laziness I suppose (patient laughs) and time; mornings can be quite rushed bang bang and off to work, evenings you get home and then you are tired, you think let’s get to bed” (P13)*
Emotional limitations that influence exercise avoidance	Lack of self-motivation and non-enjoyment of exercise	*I didn’t think they (home exercises) were time consuming, 10 min, which is not a lot of time but I think we find excuses sometimes, don’t we? (P13)*
	Preconceived opinions and experiences of little or no benefit of the intervention (physiotherapy and/or exercises)	*“I didn’t really notice any difference at all in my walking (home stretches), but I don’t really have any problems with walking” (P2)*
Physical circumstances that made exercise participation burdensome	Perceived pain by the intervention (physiotherapy and/or exercises)	*“I think it is useful to have the daily exercises, certainly, but having some physiotherapy especially on my ankles when the pressure was put on my ankles by the physio, that tended to be quite painful – so that was one of the reasons why I tended to protect myself” (P6)*
	Perceived side-effects from existing comorbidities influence the exercise frequency	*“My walking has been very difficult because of my stenosis … It was all rather put off because of my stenosis which was getting in the way; up until my stenosis got really bad, the exercises were helping me no end” (P5)*

### Support from others to do the exercises

Participants cited that support from family or physiotherapists was essential to motivate them to carry on with the home stretches. In the proof-of-concept RCT, the family members supporting the participants were mostly the female spouses. The spouses usually took the administrative responsibility of completing the exercise diaries. Others mentioned that family members reinforced the message that the exercises have been beneficial in terms of their walking ability and distance; as one participant mentioned: *“I may miss couple of sessions or a day but my wife constantly reminds me that I need to do my exercises, not to let it go back to what it was … my wife kept the diary, I did the exercises, once I done them my wife will then update and tick them off”* (P10). Other participants felt the support they received from the physiotherapists whilst attending their weekly appointments was enough to encourage them to carry on with the home stretches. Physiotherapists usually promote exercise adherence by means of developing good rapport and a trusting relationship with their patients [14], like one participant mentioned:

*“The approach of your department motivated me to cooperate because you were doing the best for me, yes for research, but you were doing the best for me”* (P3). Even though, the physiotherapist’s primary role was to deliver the intervention (foot and ankle mobilisations), some participants cited that the physiotherapist attentiveness and approachable manner meant that they felt more cooperative with the home stretches.

### Psychological factors to motivate exercise adherence

Participants reported that by doing the home stretches or receiving the physiotherapy intervention, they felt a sense of moral obligation to do the exercises. They did not want to waste time or valuable research resources, as mentioned:

*“Basically, if you are taking part in a study and they give you some exercises to do, you do them. Otherwise, it is making the entire programme a waste of time, that’s why I kept on doing it”* (P9). Some participants felt a sense of altruism and selflessness that powered them on to do the exercises with the hope that the results of this study will contribute to the knowledge base of the future management of people with diabetes: *“I suppose I was glad I was able to help with the possibility to make things better for someone else, and possibly me”* (P2)*.* Participants felt more confident and reported improvements in independence as a result of taking part in the study. This was reflected in alterations in behaviour such as not using or altered use of a walking stick: “*I feel better for taking part, I wanted to improve my ankle movements, to walk or stand. I don’t use the stick for walking is purely for my balance now, I don’t use it for weight anymore”* (P14)*.* It is unclear, however, if this was due to the intervention or a placebo effect reflected by their initial desire to improve their mobility and general well-being.

### Physical factors contributing to exercise adherence

Half of the participants reported that the home stretches improved their mobility and sense of flexibility in their foot and ankle joints. Participants repeatedly expressed this as “*freed up”* (P12) and “*stiffness eased off”* (P14) by performing the stretches on a regular basis. The physiotherapy intervention also played a role towards improving exercise adherence; participants described that following the intervention they felt “*less tight”* (P14) and “*more supple”* (P4). Participants also described how home exercises and physiotherapy reduced their pain experiences and made their symptoms more manageable: *“Particularly on my foot, my big toe was very very painful, by having the physiotherapy and doing the exercises by the wall that really helped; and one of the movements the physiotherapist was doing was unbelievable, he really manipulated that toe and it really made it better”* (P11). Also, many participants reported that the combination of home exercises and physiotherapy gave them the ability to move and function better whilst being more able to conduct activities of daily living such as “*walking up the stairs without hanging on”* (P5), “*picking things from the floor”* (P10) and “*walking further”* (P7).

### Acceptability of home exercises during and beyond the study

Many participants found the instructions for stretches easy to understand, “*not challenging”* (P7) and “*straightforward”* (P11) whilst filling in the exercise diary by “*ticking them off”* (P11) a fast and effective approach to document their progress. Participants also felt that the duration spent doing the home stretches was ideal (maximum duration 6 mins per day), which encouraged their engagement with the intervention and study, as reflected by: *“I was very happy with them because they didn’t take too long to complete (home stretches), if they had been longer I wouldn’t have stuck to the programme”* (P5). Some participants also commented that they enjoyed the home stretches and *“looked forward to doing them”* (P7). Many participants felt confident they will continue the stretches beyond the study period as they felt the stretches did “*more good than harm”* (P14).

### Social factors that contributed to exercise disengagement

Some participants felt that their lifestyle, such as *“work commitments”* (P13), *“hospital appointments and volunteering in other projects”* (P16), were reasons why they could not carry out the home stretches as often as they would have liked, as one participant stated: *“I guess things overtook when I was busy on that day, what appointments I had, if I went out early or during the day I might forget to do them and then coming back forget to do them and missed the rhythm of it”* (P6).

### Emotional limitations that influence exercise avoidance

Some participants mentioned that they lacked the motivation to do the home exercises: *“Because when I am sitting down in the evening, comfortably in front of the TV watching something, I am not going to get up to do them”* (P4). A portion of participants mentioned fear of injury as the predominant factor of stopping them do the stretches: *“I wanted to find out whether it was going to be of benefit to me (exercises) and whether it was going to harm the feelings that I had in my feet, because I am very cautious, in my movement, because I am aware of my movement if you understand what I mean, I am very protective about the exercises that I do”* (P6). Furthermore, some participants assumed that exercises and/or physiotherapy will not change their foot and ankle movements or be beneficial to them and therefore, they did not fully engage with them: *“I didn’t find it made any difference (physiotherapy) to my movements whatsoever, but I didn’t expect it to be honest … because I am already doing more than the physiotherapy for myself already”* (P9).

### Physical circumstances that made exercise participation burdensome

A number of participants expressed side effects either from the home stretches or the foot and ankle mobilisations, which led to poor adherence: *“Well you knew you done them, no pain no gain and all that, I didn’t feel lasting pain but obviously they weren’t pain free”* (P16). Side effects from existing co-morbidities “chest infection” (P12) and *“low back pain”* (P11) were also given as explanations for poor adherence to home stretches.

## Discussion

This embedded qualitative study in a proof-of-concept RCT aimed to explore the participants’ experiences of an intervention related to home stretches. One of the factors that positively influenced adherence to the requested intervention was the attendance of weekly appointments for the mobilisations of foot and ankle and the return of the exercise diaries. Encouragement by healthcare professionals and family support is a common denominator for facilitating exercise behaviours [1, 22] whereas lacking the support from family or friends tends to be a barrier to exercise [24]. Social support could also be rebranded as an “affective intervention” which seeks to enhance adherence by providing emotional support and encouragement or by helping people adhere to behavioural changes by building rapport [15, 44]. In our study, we found that both family members and the physiotherapist provided this means of emotive support.

Another theme that facilitated exercise adherence was the sense of duty and moral obligation. Our participants volunteered to take part in the study and perhaps the majority of them felt the intrinsic motivation and driving force to do the exercises in order to help others (altruistic volunteerism) and/or themselves (egoistic volunteerism) [34, 48]. The relationship between altruism and the different functions of volunteerism is complex and addressed in the literature [31, 46, 47] including the potential health and wellbeing benefits of those who volunteer [3]. These benefits of wellbeing were also expressed by our participants who felt that the home stretches improved their confidence and perceived improvements in their balance [8].

Another theme was the self-perceived physical improvements in terms of reducing stiffness and pain experiences in the foot and ankle following the intervention. To our knowledge, no studies have been published investigating the effects of mobilisations and home stretches on participants’ self-perceived foot and ankle stiffness and/or pain scores. Earlier studies have carried interventions, which included a combination of foot stretches and strengthening exercises on the effect of foot and ankle ROM and function, and have found improvements in these [7, 27, 42]. A pilot study reported that 20 sessions of physiotherapy on ankle, subtalar, midtarsal and foot joints resulted in near-normal joint mobility but these changes were not long lasting [10]. The aforementioned studies measured joint mobility with objective measures. Direct comparisons between studies cannot be made due to the different methodological approaches used. Differences are found in the duration, type and dosage of home exercise program. Our study supports the preliminary argument that mobilisations and home stretches could play an important factor in improving foot and ankle ROM in people with diabetes. This is reinforced by the existing ankle mobilisation literature, focusing on patients with chronic ankle instability, where a positive association between mobilisations and increases in ankle ROM has been established [13, 17, 18].

In our study, participants mentioned that the instructions and exercise diary was easy to complete, that the home stretches did not take long and some participants also mentioned the willingness to continue with the exercises beyond the study. The intervention of the proof-of-concept RCT was indeed short and easy to do in a home-based setting. According to Schechter and Walker [44] exercise diaries can be used as a memory aid, reminder or a way to self-monitor and therefore diaries can be used as a behavioural approach model of interventions to improve adherence rates. However, self-reports can sometimes provide overestimates of adherence as some patients tend to report higher levels of adherence in order to please the healthcare providers or avoiding embarrassment [44].

Factors influencing exercise non-adherence in our study seem to be in agreement with the literature. Lack of time and motivation were some of the main reasons that contributed to poor adherence [1, 4, 9, 50]. Some participants had preconceived ideas that the exercises would not be beneficial and influenced their decision-making. This is in with [4],who also reported negative perceptions held by participants as a barrier to exercise by [4]. The existence of co-morbidities were also factors that negatively influenced exercise adherence. This has been described by Lim et al. [24] reporting medical conditions as factors for hindering exercise adherence [24].

### Limitations

A limitation of the study is the nature of qualitative research methodologies and its generalisability. Our sample of participants, which may differ to the wider population of people with diabetes in the UK, was small and all participants were taking part in the intervention arm of our proof-of-concept RCT. Therefore, our findings might not be representative of the wider population of people with diabetes. Another consideration is the acceptability of the intervention. In our study, the results demonstrated that the intervention was well received by most participants. However, we cannot rule out self-selection bias, whereas the more sceptic participants could have declined to participate in our PoC RCT.

Only two of the sixteen participants in our study were female. There is some research to suggest that women are less likely to participate in clinical trials [26] and this disproportionate ratio between male:female was also reflected in the proof-of-concept RCT: only 16.5% of participants were female. Another factor to consider was the timing of the interview that took place following the RCT’s outcome measure collection session. It could be argued that some participants had experienced fatigue which subsequently affected their willingness to elaborate in their responses during the interview process. Lastly, the chief investigator could have introduced some interviewer bias due to his own personal interest in the research. However, in order to maintain objectivity of the data, this was analysed with another member of the team experienced in qualitative research (JML).

## Conclusion

Our study indicates that the intervention of home stretches combined with foot and ankle mobilisations was acceptable to the participants. The findings suggest that good levels of exercise adherence can be achieved when including physiotherapy treatment and weekly exercise diaries. A clinician who demonstrates the exercises and checks how the patient is finding them (perceived difficulty in execution and discomfort), combined with an exercise diary, can be a motivating factor to improve long-term exercise adherence. Further research is needed to understand factors influencing adherence of patients from diverse backgrounds to design supportive interventions that improve ROM and reductions in foot and ankle pain. A full-scale RCT is warranted to investigate the benefits of exercises and physiotherapy in the management of the diabetic foot.

## Supplementary Information

**Additional file 1.** Home Exercise Programme.

**Additional file 2.** Foot and Ankle Mobilisation in Diabetic Peripheral Neuropathy.

**Additional file 3.** Interview guide.

**Additional file 4.** COREQ (COnsolidated criteria for REporting Qualitative research) Checklist.

**Additional file 5.** Point-by-point response letter.

## Data Availability

All anonymised data can be made available from the correspondng author upon reasonable request.

## Abbreviations

PoC: Proof-of-Concept
RCT: Randomised controlled trial
ROM: Range of movement
BMI: Body mass index

## References

[1] Advika TS, Idiculla J, Kumari SJ (2017). Exercise in patients with type 2 diabetes: facilitators and barriers - A qualitative study. J Fam Med Prim Care.

[2] Bailey DL, Holden MA, Foster NE, Quicke JG, Haywood KL, Bishop A (2020). Defining adherence to therapeutic exercise for musculoskeletal pain: a systematic review. Br J Sports Med.

[3] Black W, Living R (2004). Volunteerism as an occupation and its relationship to health and wellbeing. Br J Occup Ther.

[4] Booth AO, Lowis C, Dean M, Hunter SJ, McKinley MC (2012). Diet and physical activity in the self-management of type 2 diabetes: barriers and facilitators identified by patients and health professionals. Prim Health Care Res Dev.

[5] Boulé NG, Haddad E, Kenny GP, Wells GA, Sigal RJ (2001). Effects of exercise on glycemic control and body mass in type 2 diabetes mellitus: a meta-analysis of controlled clinical trials. JAMA.

[6] Braun V, Clarke V (2006). Using thematic analysis in psychology. Qual Res Psychol.

[7] Cerrahoglu L, Kosan U, Sirin TC, Ulusoy A (2016). Range of motion and plantar pressure evaluation for the effects of self-care foot exercises on diabetic patients with and without neuropathy. J Am Podiatr Med Assoc.

[8] Cyarto EV, Brown WJ, Marshall AL, Trost SG (2008). Comparative effects of home- and group-based exercise on balance confidence and balance ability in older adults: cluster randomized trial. Gerontology.

[9] Dave D, Soni S, Irani A (2015). Identification of barriers for adherence to exercise in type 2 diabetes mellitus--a cross sectional observational study. Physiotherapy.

[10] Dijs HM, Roofthooft JM, Driessens MF, De Bock PG, Jacobs C, Van Acker KL (2000). Effect of physical therapy on limited joint mobility in the diabetic foot. A pilot study. J Am Podiatr Med Assoc.

[11] Dunstan DW, Vulikh E, Owen N, Jolley D, Shaw J, Zimmet P (2006). Community center-based resistance training for the maintenance of glycemic control in adults with type 2 diabetes. Diabetes Care.

[12] Frost R, McClurg D, Brady M, Williams B (2016). Optimising the validity and completion of adherence diaries: a multiple case study and randomised crossover trial. Trials.

[13] Fujii M, Suzuki D, Uchiyama E, Muraki T, Teramoto A, Aoki M, Miyamoto S (2010). Does distal tibiofibular joint mobilization decrease limitation of ankle dorsiflexion?. Man Ther.

[14] Harman K, Bassett R, Fenety A, Hoens AM (2011). Client education: communicative interaction between physiotherapists and clients with subacute low Back pain in private practice. Physiother Can.

[15] Hartley S (2016). Gateway to health: physiotherapists’ role in promoting the physical and mental wellbeing of people with long-term conditions. Physiotherapy.

[16] Hayes C, Kriska A (2008). Role of physical activity in diabetes management and prevention. J Am Diet Assoc.

[17] Hoch MC, McKeon PO (2011). Joint mobilization improves spatiotemporal postural control and range of motion in those with chronic ankle instability. J Orthop Res.

[18] Hoch MC, Mullineaux DR, Andreatta RD, English RA, Medina-McKeon JM, Mattacola CG, McKeon PO (2014). Effect of a 2-week joint mobilization intervention on single-limb balance and ankle arthrokinematics in those with chronic ankle instability. J Sport Rehabil.

[19] Kästenbauer T, Sauseng S, Sokol G, Auinger M, Irsigler K (2001). A prospective study of predictors for foot ulceration in type 2 diabetes. J Am Podiatr Med Assoc.

[20] Kerr M, Rayman G, Jeffcoate WJ (2014). Cost of diabetic foot disease to the National Health Service in England. Diabet Med.

[21] Knowler WC, Barrett-Connor E, Fowler SE, Hamman RF, Lachin JM, Walker EA, Nathan DM (2002). Reduction in the incidence of type 2 diabetes with lifestyle intervention or metformin. N Engl J Med.

[22] Laranjo L, Neves AL, Costa A, Ribeiro RT, Couto L, Sá AB (2015). Facilitators, barriers and expectations in the self-management of type 2 diabetes—a qualitative study from Portugal. Eur J Gen Pract.

[23] Lavery LA, Armstrong DG, Boulton AJ (2002). Ankle equinus deformity and its relationship to high plantar pressure in a large population with diabetes mellitus. J Am Podiatr Med Assoc.

[24] Lim RBT, Wee WK, For WC, Ananthanarayanan JA, Soh YH, Goh LML, Tham DKT, Wong ML (2020). Correlates, facilitators and barriers of physical activity among primary care patients with prediabetes in Singapore – a mixed methods approach. BMC Public Health.

[25] Littlewood C, Mawson S, May S, Walters S (2015). Understanding the barriers and enablers to implementation of a self-managed exercise intervention: a qualitative study. Physiotherapy.

[26] Meinert CL, Gilpin AK, Unalp A, Dawson C (2000). Gender representation in trials. Control Clin Trials.

[27] Monteiro RL, Ferreira J, Silva EQ, Donini A, Cruvinel-Júnior RH, Verissímo JL, Bus SA, Sacco ICN (2020). Feasibility and preliminary efficacy of a foot-ankle exercise program aiming to improve foot-ankle functionality and gait biomechanics in people with diabetic neuropathy: A randomized controlled trial. Sensors (Basel).

[28] Mueller MJ, Sinacore DR, Hastings MK, Strube MJ, Johnson JE (2003). Effect of Achilles tendon lengthening on neuropathic plantar ulcers. A randomized clinical trial. J Bone Joint Surg Am.

[29] Mueller MJ, Zou D, Lott DJ (2005). “pressure gradient” as an indicator of plantar skin injury. Diabetes Care.

[30] Nelson KM, Reiber G, Boyko EJ (2002). Diet and exercise among adults with type 2 diabetes: findings from the third national health and nutrition examination survey (NHANES III). Diabetes Care.

[31] Omoto AM, Snyder M (2002). Considerations of community:the context and process of volunteerism. Am Behav Sci.

[32] Palinkas LA, Horwitz SM, Green CA, Wisdom JP, Duan N, Hoagwood K (2015). Purposeful sampling for qualitative data collection and analysis in mixed method implementation research. Admin Pol Ment Health.

[33] Pham H, Armstrong DG, Harvey C, Harkless LB, Giurini JM, Veves A (2000). Screening techniques to identify people at high risk for diabetic foot ulceration: a prospective multicenter trial. Diabetes Care.

[34] Phillips M (1982). Motivation and expectation in successful volunteerism. J Volunt Action Res.

[35] Praet SF, van Loon LJ (2009). Exercise therapy in type 2 diabetes. Acta Diabetol.

[36] Praet SF, van Rooij ES, Wijtvliet A, Boonman-de Winter LJ, Enneking T, Kuipers H, Stehouwer CD, van Loon LJ (2008). Brisk walking compared with an individualised medical fitness programme for patients with type 2 diabetes: a randomised controlled trial. Diabetologia.

[37] Ritchie J, Lewis J, Elam G (2011). Designing and selecting samples. Qualitative Research Practice.

[38] Robbins JM, Strauss G, Aron D, Long J, Kuba J, Kaplan Y (2008). Mortality rates and diabetic foot ulcers: is it time to communicate mortality risk to patients with diabetic foot ulceration?. J Am Podiatr Med Assoc.

[39] Sacco IC, Hamamoto AN, Gomes AA, Onodera AN, Hirata RP, Hennig EM (2009). Role of ankle mobility in foot rollover during gait in individuals with diabetic neuropathy. Clin Biomech (Bristol, Avon).

[40] Sacco IC, Sartor CD (2016). From treatment to preventive actions: improving function in patients with diabetic polyneuropathy. Diabetes Metab Res Rev.

[41] Salsich GB, Mueller MJ, Hastings MK, Sinacore DR, Strube MJ, Johnson JE (2005). Effect of Achilles tendon lengthening on ankle muscle performance in people with diabetes mellitus and a neuropathic plantar ulcer. Phys Ther.

[42] Sartor CD, Hasue RH, Cacciari LP, Butugan MK, Watari R, Pássaro AC, Giacomozzi C, Sacco IC (2014). Effects of strengthening, stretching and functional training on foot function in patients with diabetic neuropathy: results of a randomized controlled trial. BMC Musculoskelet Disord.

[43] Sartor CD, Watari R, Passaro AC, Picon AP, Hasue RH, Sacco ICN (2012). Effects of a combined strengthening, stretching and functional training program versus usual-care on gait biomechanics and foot function for diabetic neuropathy: a randomized controlled trial. BMC Musculoskelet Disord.

[44] Schechter CB, Walker EA (2002). Improving adherence to diabetes self-management recommendations. Diabetes Spectr.

[45] Shultz JA, Sprague MA, Branen LJ, Lambeth S (2001). A comparison of views of individuals with type 2 diabetes mellitus and diabetes educators about barriers to diet and exercise. J Health Commun.

[46] Smith DH (1981). Altruism, Volunteers, and Volunteerism. J Volunt Action Res.

[47] Snyder M, Omoto AM (2008). Volunteerism: social issues perspectives and social policy implications. Soc Issues Policy Rev.

[48] Steen T (2006). Public sector motivation: is there something to learn from the study of volunteerism?. Public Pol Adm.

[49] Terada M, Pietrosimone BG, Gribble PA (2013). Therapeutic interventions for increasing ankle dorsiflexion after ankle sprain: a systematic review. J Athl Train.

[50] Thomas N, Alder E, Leese GP (2004). Barriers to physical activity in patients with diabetes. Postgrad Med J.

[51] Tong A, Sainsbury P, Craig J (2007). Consolidated criteria for reporting qualitative research (COREQ): a 32-item checklist for interviews and focus groups. Int J Qual Health Care.

[52] van der Wees PJ, Lenssen AF, Hendriks EJM, Stomp DJ, Dekker J, de Bie RA (2006). Effectiveness of exercise therapy and manual mobilisation in acute ankle sprain and functional instability: a systematic review. Aust J Physiother.

[53] Young R, Nix S, Wholohan A, Bradhurst R, Reed L (2013). Interventions for increasing ankle joint dorsiflexion: a systematic review and meta-analysis. J Foot Ankle Res.

