# Supplementary material for: Experiences of foot and ankle mobilisations combined with home stretches in people with diabetes: a qualitative study embedded in a proof-of-concept randomised controlled trial
Source: J Foot Ankle Res. 2022 Jan 29;15:7. doi: 10.1186/s13047-022-00512-z (PMC8801130; doi:10.1186/s13047-022-00512-z)
Supplement: Supplementary file 2 — Additional file 2. Foot and Ankle Mobilisation in Diabetic Peripheral Neuropathy. [file 13047_2022_512_MOESM2_ESM.docx]

Foot and Ankle Mobilisation in Diabetic Peripheral Neuropathy

Participant Exercise Diary

Participant Number

**Exercise 1** **Exercise 2** **Exercise 3**


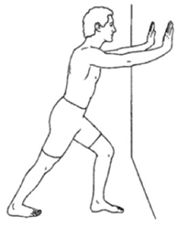

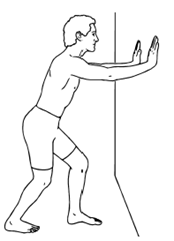

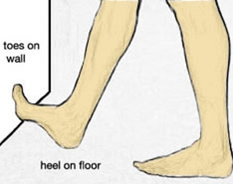


Welcome! Thank you once again for volunteering for this study. As part of the study we would like you to complete this diary every day.

**Exercise aims**: These exercises aim to increase the movement in your foot and ankle. We expect a more mobile foot and ankle will be associated with lower loads under your foot while walking and lead to a reduced risk of foot ulcers. It may also make walking easier.

On each day we would like you to tick ☑ which exercises you have done. This includes the three exercises prescribed by your therapist which are numbered in your home exercise sheet (quick reference at the front of this pack). In the next column we would like you to indicate the volume of exercises by ticking ☑ if you have done them. If you were not able to complete the prescribed amount of exercises, use the “comments” box to inform your therapist how much you have exercised. In the end of this booklet, you will find two questions. Answering these will give us valuable information to understand what enabled you or stopped you from carrying out these exercises.

It is important to be as accurate as you can but don’t worry if you cannot exercise every day. Your treating therapist will collect your exercise diary weekly so it is important to remember to bring this with you on each appointment. You will collect a new diary by your therapist each week.

In the meantime if you have any questions please contact:

Jon Marsden 01752 587 590 [jonathan.marsden@plymouth.ac.uk](mailto:jonathan.marsden@plymouth.ac.uk) (mon-friday)

A large print version of this booklet is available on request

Tick each exercise as appropriately ☑


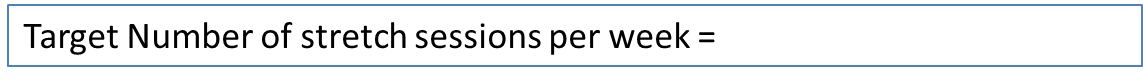


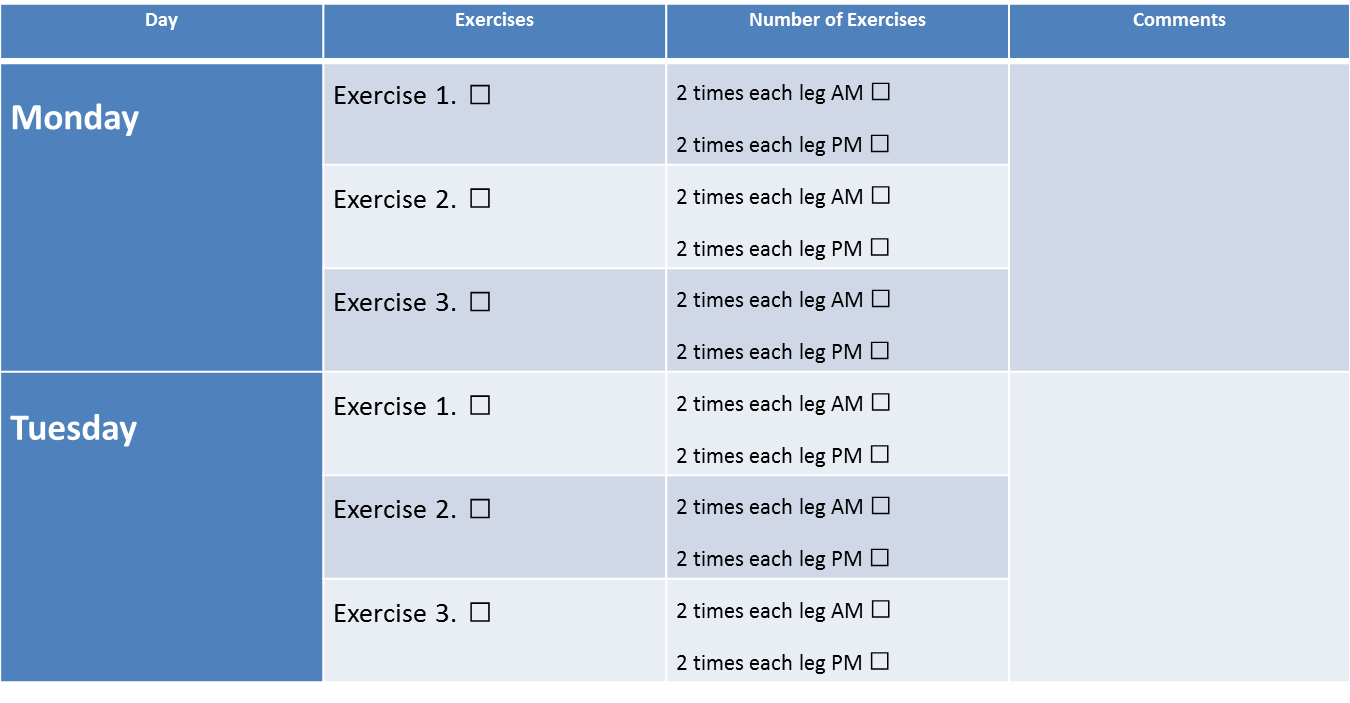


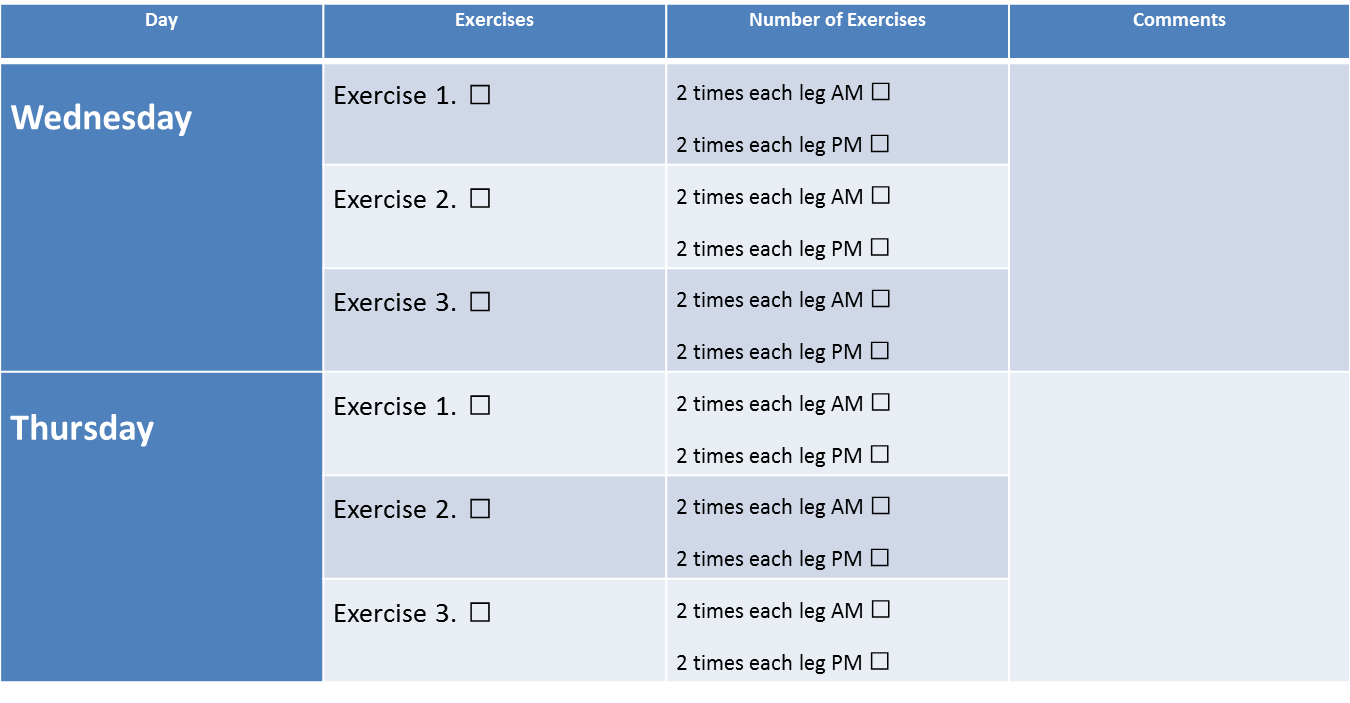


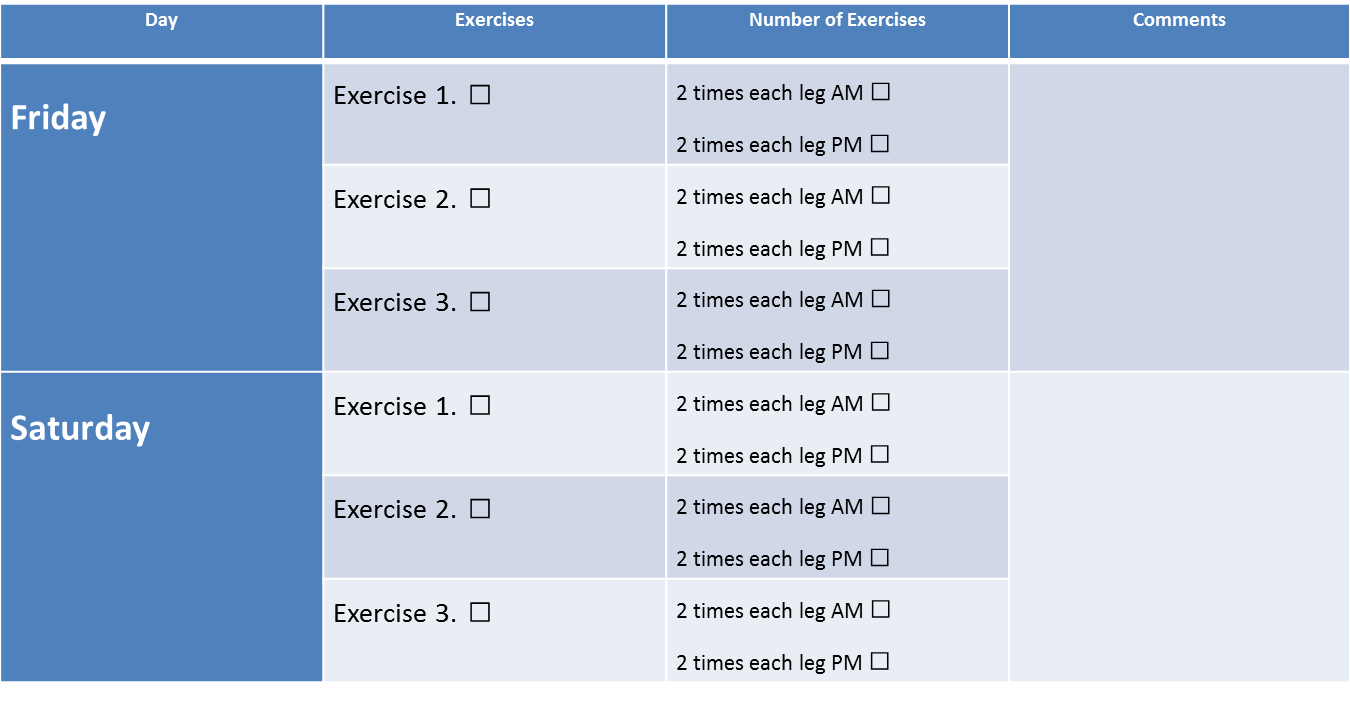


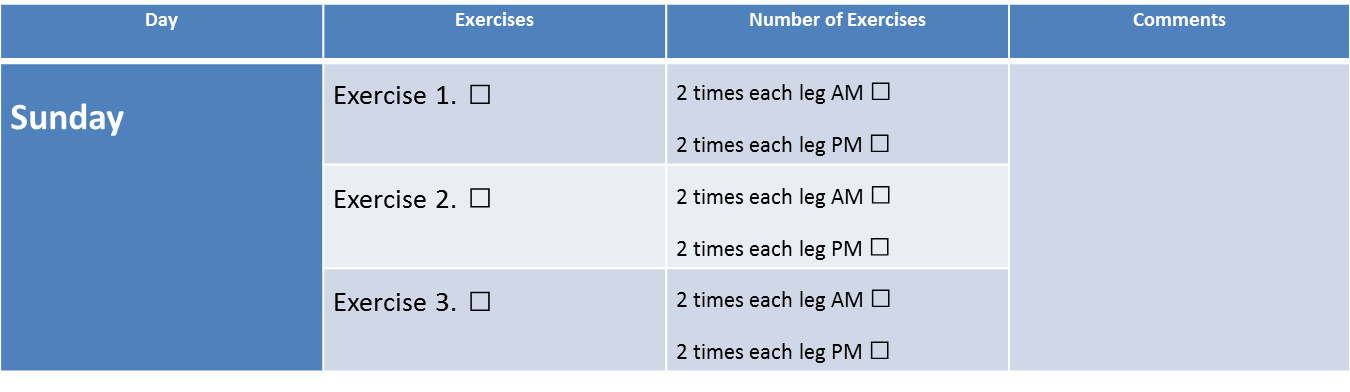


Congratulations, you have completed your first week of exercises!


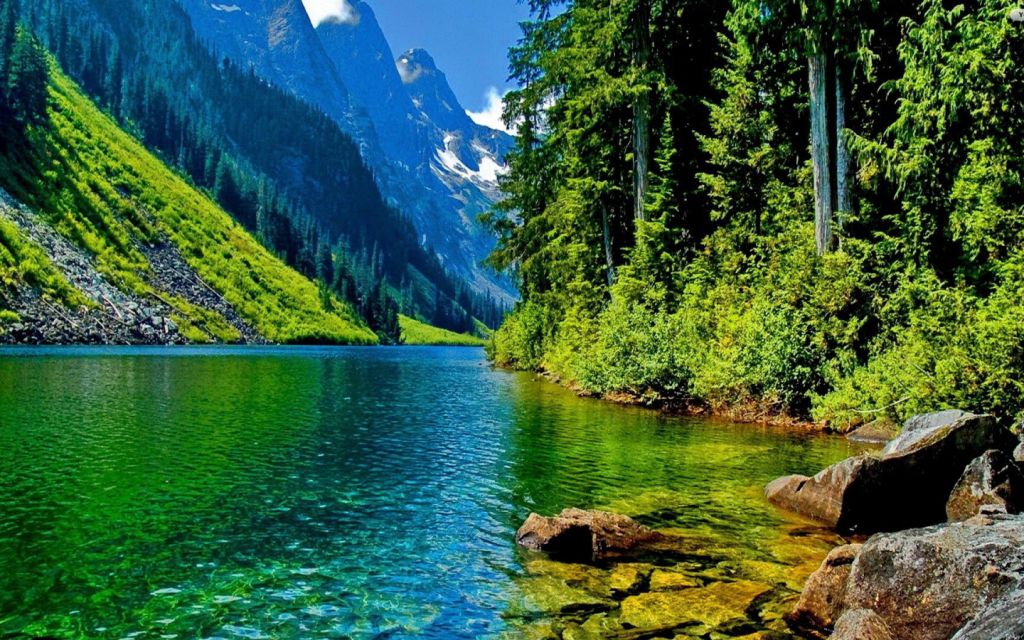


| Question 1: What helped you to decide **to do** the exercises this week? (For example: it might improve your walking or ability to stand) |
| --- |
|  |

| Question 2: What **stopped** you from doing the exercises this week? (For example: lack of time, couldn’t be bothered, physical pain in muscles or joints from doing the exercises) |
| --- |
|  |

Thank you for taking the time to complete this diary
