# Supplementary material for: Experiences of foot and ankle mobilisations combined with home stretches in people with diabetes: a qualitative study embedded in a proof-of-concept randomised controlled trial
Source: J Foot Ankle Res. 2022 Jan 29;15:7. doi: 10.1186/s13047-022-00512-z (PMC8801130; doi:10.1186/s13047-022-00512-z)
Supplement: Supplementary file 3 — Additional file 3. Interview guide. [file 13047_2022_512_MOESM3_ESM.docx]

Additional File 3. Interview guide

| 1. **I would like to explore your experiences of participating in our study and doing the home exercise programme**     - 1. How did you find taking part in the study?      2. Tell me about your thoughts and feelings that influenced your decision whether or not (*depending on group allocation)* to carry out the stretches at home?         1. Suggested prompt questions (depending on group allocation):            1. What helped you decide to do the exercises (i.e. improve walking, standing)            2. What stopped you from doing the exercises (i.e. lack of time, pain, fatigue)      3. How did you find the home exercise programme?         1. Suggested prompt questions:            1. Can you explain a bit more about xxx?      4. Could anything be changed about the home exercise programme?         1. Prompt questions:            1. Can you tell me why no (or why yes)?      5. Did you find the exercise diary easy to fill in?      6. Did you find the questions in the end of the exercise diary easy to understand?      7. In your opinion, how would you rate your overall satisfaction with:  \|  \| Very good \| Good \| OK \| Poor \| Very poor \| \| --- \| --- \| --- \| --- \| --- \| --- \| \| Participating in the trial \|  \|  \|  \|  \|  \| \| Outcome measure sessions \|  \|  \|  \|  \|  \| \| Understanding of information sheets \|  \|  \|  \|  \|  \|   Can you please explain your answers?   1. **Overall**    1. In your opinion, is there anything you would change about the home exercise program?    2. In your opinion, could you identify any factors that could make a future trial easier to perform? |
| --- | --- | --- | --- | --- | --- | --- | --- | --- | --- | --- | --- | --- | --- | --- | --- | --- | --- | --- | --- | --- | --- | --- | --- | --- |
