# Supplementary material for: Experiences of foot and ankle mobilisations combined with home stretches in people with diabetes: a qualitative study embedded in a proof-of-concept randomised controlled trial
Source: J Foot Ankle Res. 2022 Jan 29;15:7. doi: 10.1186/s13047-022-00512-z (PMC8801130; doi:10.1186/s13047-022-00512-z)
Supplement: Supplementary file 5 — Additional file 5. Point-by-point response letter. [file 13047_2022_512_MOESM5_ESM.docx]

Point-by-point response letter

Dear Dr Hendry

Thank you for accepting my revisions and the constructive feedback for some further revisions to be made. We like to apologise for missing the original comments made by reviewer 2. We believe that our responses to the comments made by Reviewer 2 have further improved the clarity of our paper.

Attached below are our responses. We have also highlighted with ‘track changes’ all the revisions made in the main manuscript.

On behalf of the authors

Yours sincerely,

Vasileios Lepesis

Corresponding author

**Reviewer 1**

**I thank the Authors for revisions. I have no further comments. Congratulations, great work and good luck for the future.**

Response: Thank you for your original suggestions and accepting our revisions.

**Reviewer 2**

I thank the Authors for being responsive to my questions on the Abstract and Background. I just have one minor comment on this:

**p.6. line 2-4. "Adherence to exercise programmes in the diabetic population varies between 10 and 80% (Praet & van Loon, 2009)."
The term "diabetic" is usually avoided.**

Response: We have changed the term “diabetic” to “people with diabetes” (line 21, p.5)

However, answers to my original comments on the Methods, Findings, Discussion, etc. are missing. I copy them in here below:

**METHODS
p.6, line 2-7. The COnsolidated criteria for REporting Qualitative research checklist has been used to report the findings of this study (27). Change to past tense?**

Response: Thank you for your comment. We have changed this to: “The Consolidated criteria for Reporting qualitative research was used to report the findings…” (lines 16-18, p.6)

**p.6, line 41. "The selection of study participants was based on the purposive sampling
strategy (29)." Please specify what participant characteristics (gender, age, diabetes type, etc.) that were considered in the sampling (was the aim heterogeneity?).**

Response: Thank you for your comment and question. You are correct in thinking that participants were selected with heterogeneity/variation in mind and this was primarily based on the factor of adherence (or not) to the prescribed home stretches. Subsequently, only participants who received the intervention (i.e. foot and ankle mobilisations combined with home stretches) were selected for the semi-structured interviews.

We added this sentence to clarify further: “Participants were identified and selected based on their experiences to the intervention with the aim of capturing a wide range of perspectives relating to the prescribed home stretches”. (lines 8-10, p.7)

**p.6, line 44. "Of the 32 participants in the intervention group, 16 participants were
recruited providing adequate levels of data saturation (30)." Please specify how data saturation was assessed.**

Response: Thank you for your question. We view data saturation as a process and a ‘matter of degree’ rather than a specific, discrete point in time at which saturation was achieved. We have included this additional information to the sentence to make this clear: “During the analyses of the final two interviews, we experienced that no new main themes emerged, hence the authors assumed that adequate levels of data saturation was achieved (lines 18-21, p.7)

**p.6, line 58. I would suggest to move the link to Clinical trials to earlier in the text when describing the RCT.**

Response: Thank you for your suggestion. We have moved the link to the Clinical trials earlier in the text when the RCT was first described: “Participants were recruited as part of the intervention group of a PoC RCT (<https://clinicaltrials.gov/ct2/show/NCT03195855>). (line 24,p.5)

**p.7, line 53. "The third step (theme searching) carried out by two researchers (VL, JML) involved the interpretive stages of the data by collating codes into sub-themes." Are commas missing here?**

Response: Thank you for your suggestion; a comma has been added in the sentence: “The third step (theme searching) carried out by two researchers (VL, JML), involved the interpretive stages of the data by collating codes into sub-themes” (line 1, p.9)

**p.8, line 22. I suggest to add the interviewer's initials in parentheses.**
Response: Thank you for your comment. This was addressed in the earlier revision. The interviewer’s initials have been added in parentheses: “The chief investigator of the PoC RCT was also the interviewer (VL)” (line 12, p.9)

**p.8, line 36. "The interviewer was blinded to the group of exercise the participants' belonged to." Were not all participants in the intervention group of the RCT? In the interview guide, it is stated "(depending on group allocation)" which would indicate that both groups were interviewed, but in the Methods, it says that all participants came from the intervention group. Please clarify.**

Response: Thank you for your questions and apologies for the confusion. We have addressed this in our previous version in the introduction section (lines 8-19, p. 6) and methods section (lines 6-8, pp.7-8). You are right in thinking that all participants in the intervention group of the RCT were prescribed with home stretches, however the interviewer was blinded to whether the participants adhered or not to the home stretches. We have included “adherent versus non-adherent group” to clarify this further (lines 18-19, p.9)  **FINDINGS**

**Table 1. Is participant 6's BMI of 62 correct (it could be, of course)?**
Response: Thank you for pointing this out. We checked our records of participant characteristics and this patient’s weight was 150kgs and his height 155cms.

**p.8, line 9. "6) Psychological self-restraints that influence exercise avoidance". Consider clarifying direction of influence.**

Response: Thank you for your comment. This was also addressed by other reviewers and we had changed this already in the first revision and the new theme title is: “Emotional limitations that influence exercise avoidance” (lines 11-12, 10)
 **p.11, line 29. Quotation mark is missing in the beginning of the quote.**

Response: Thank you for pointing this out. A quotation mark has been added: “*I feel better for taking part, I wanted to improve my ankle movements, to walk or stand. I don’t use the stick for walking is purely for my balance now, I don’t use it for weight anymore”* (P14) (line 23, p.11) **p.11, line 51. "The physiotherapy intervention also played a role towards improving exercise adherence; participants described that following the intervention they felt "less tight" (P14) and "more supple" (P4)." The quotes do not seem to support the interpretation, that is, that the intervention improved adherence, but rather described positive experiences of the effect.**

Response: Thank you for your feedback. Based on the experience of a number of participants, having foot and ankle mobilisations (physiotherapy) contributed to their perceptions of reduction in big toe and ankle joint restriction. Subsequently, these participants felt more motivated to carry out their home stretches in order to maintain the feeling of flexibility in these joints. Therefore, we believe that physiotherapy played an essential role in maintaining or improving the motivation of exercise adherence for these participants.  **p.13, line 41. Please review the section "Physical circumstances that made exercise participation burdensome" for consistency. The heading states that physical circumstances made exercise burdensome, without any interpretation whether or not this affected adherence. The first quote seems to describe a participant who experienced pain but still performed the exercises, that is, showed high adherence. In contrast, the next sentence "Side effects from existing co-morbidities "chest infection" (P12) and "low back pain" (P11) were also given as explanations for poor adherence to home stretches" describes a scenario where participants explain poor adherence in terms of physical circumstances.**

Response: Thank you for your feedback and the need to clarify this further. The theme of “Physical circumstances that made exercise participation burdensome" describes reasons for poor adherence to the home stretches. The first quote included in this section is indeed a bit misleading so we removed part of it since this participant did not adhere fully to the home stretches due to pain. This section now reads as follows: “A number of participants expressed side effects either from the home stretches or the foot and ankle mobilisations, which led to poor adherence: *“Well you knew you done them, no pain no gain and all that, I didn’t feel lasting pain but obviously they weren’t pain free” (P16), (lines 5-8, p.14)* **DISCUSSION
p.14, line 45. "The relationship between altruism and the different functions of volunteerism is complex and has been address in the literature" Change to "addressed"?**

Response: Thank you for your suggestion. This has been altered: “The relationship between altruism and the different functions of volunteerism is complex and addressed in the literature” (line 6, p.15) **p.14, line 56 - p.15, line 30. I find it a bit difficult to understand where the argument is going in this section. Please review it to see if the line of argument can be clarified.
p.14, line 56. "Another theme was the self-perceived physical improvements in terms of reducing stiffness and pain experiences in the foot and ankle following the intervention. To our knowledge, no studies have been published investigating the effects of mobilisations and home stretches on participants' self-perceived foot and ankle stiffness and/or pain scores."
If no studies have investigated this before, why were the results compared to other studies, or was it because the other studies measured ROM (objectively)? Does "self-perceived" only refer to stiffness as pain is always self-perceived?
Line 5. "Earlier studies have carried interventions of a combination of foot stretches and strengthening exercises on the effect of foot and ankle and have found improvements in these (47-49)." What were the effect variables in these studies?
Line 10. "A pilot study reported that 20 sessions of physiotherapy on ankle, subtalar, midtarsal and foot joints resulted in near-normal joint mobility but these changes were not long lasting (50)." What is the point the authors wish to bring to the readers' attention?
Line 15. "However, direct comparisons between studies cannot be made due to the different
methodological approaches used." I agree, it is also seems like the other studies had other diagnostic groups than in the current study.
Line 19. "Our study supports the argument that mobilisations and home stretches can be an important factor in improving foot and ankle ROM in people with diabetes." Although promising, I think these things are evaluated with more scientific rigour in the proof-of-concept RCT and future RCT where a control group is used as comparison. Therefore, it may be a bit unjustified to state this in too certain terms in this qualitative, uncontrolled study.
Line 24. "This is reinforced by the existing ankle mobilisation literature, where a positive association between mobilisations and increases in ankle ROM has been established (13, 51, 52)." Yes, but again these other studies seem to include other patient populations.**

Response: Thank you for your comments and questions. This qualitative study is embedded in our quantitative PoC RCT where data triangulation was used to support the argument of physiotherapy and home stretches with the RCT’s findings. We have reviewed and changed sections of this paragraph to make our argument easier to follow. These changes can be found in lines 12-6, p.15-16. **p.15, line 39. "The intervention of the proof-of-concept RCT was indeed short and easy to do
in a home-based setting. This might be different in trials with outdoors or longer or
aerobic" I believe the second sentence can be deleted without losing information, it does not seem so important in this context as the current study is very far from an aerobic exercise intervention.**

Response: Thank you for pointing this out. We have deleted the second sentence as suggested: lines 11-12, p.16 **p. 16, line 36-39. "In our study, the results demonstrated that the intervention was well received by most participants." This is important, but there is a risk that the most sceptic patients declined to participate in the RCT.**

Response: Thank you for your comment. We appreciate that most of our participants were highly motivated to take part in our study and they wanted to take part in order to understand the role diabetes plays when it comes to function of their feet. We acknowledge the potential for self-selection bias (or volunteer bias) by adding another sentence for clarification: “However, we cannot rule out self-selection bias, whereas the more sceptic participants could have declined to participate in our PoC RCT” (lines 13-14, p.17)
 **p. 16, line 39-44. "However, we are not able to assume that the acceptability will remain high if the intervention involved aerobic activity as recommended in primary care (56)."
It does not seem necessary to point this out, as aerobic exercise interventions seem very far from the current intervention.**

Response: Thank you for your comment. We have deleted the above sentence as suggested: lines 14-16, p.17 **CONCLUSION
Would it be possible to be more concrete and specific on the take-home-message/implications for future research and clinic? What should researchers and clinicians do to improve patient adherence?**

Response: Thank you for your suggestion. We have altered this section aiming to be more direct and a take-home-message: “A clinician who demonstrates the exercises and checks how the patient is finding them (perceived difficulty in execution and discomfort), combined with an exercise diary, can be a motivating factor to improve long term exercise adherence. Further research is needed to understand factors influencing adherence of patients from diverse backgrounds to design supportive interventions that improve ROM and reductions in foot and ankle pain is also needed” (lines 7-12, pp.18) **GENERAL
The abbreviation PPP seems superfluous as it is only used once after introduced on p.1.**

Response: The PPP abbreviation has been deleted and the PoC (Proof-of-Concept) added (line 19, p.18).

**Looking at the interview guide, there are a few quantitative questions as well. Have the authors consider to report these quantitative results too?**

Response: Thank you for your comment. The quantitative results of the interviews have been reported as part of the quantitative PoC RCT study**.

REFERENCES
On a few places, there seem to be a mix of Harvard and Vancouver systems. Please check p. 4, line 44, p.14, line 44, and p.16, line 10.**

Response: Thank you for pointing this out. We have re-assessed the manuscript and corrected the errors.
